# Black bears alter movements in response to anthropogenic features with time of day and season

**DOI:** 10.1186/s40462-019-0166-4

**Published:** 2019-07-11

**Authors:** Katherine A. Zeller, David W. Wattles, Laura Conlee, Stephen DeStefano

**Affiliations:** 1 Massachusetts Cooperative Fish and Wildlife Research Unit, University of Massachusetts, 160 Holdsworth Way, Amherst, MA 01003 USA; 2 Massachusetts Division of Fisheries and Wildlife, Westborough, MA USA; 3 Missouri Department of Conservation, Columbia, MO USA; 4 U.S. Geological Survey, Massachusetts Cooperative Fish and Wildlife Research Unit, University of Massachusetts, Amherst, MA USA

**Keywords:** Carnivore, Conservation, Movement ecology, Human development, Multi-scale habitat selection, Massachusetts

## Abstract

**Background:**

With the growth and expansion of human development, large mammals will increasingly encounter humans, elevating the likelihood of human-wildlife conflicts. Understanding the behavior and movement of large mammals, particularly around human development, is important for crafting effective conservation and management plans for these species.

**Methods:**

We used GPS collar data from American black bears (*Ursus americanus*) to determine how seasonal food resources and human development affected bear movement patterns and resource use across the Commonwealth of Massachusetts.

**Results:**

We found that though bears moved more and avoided human development during crepuscular and daylight hours than at night, bears preferentially moved through human dominated areas at night. This indicates bears were mitigating the risk of human development by altering their behavior to exploit these areas when human activity is low. This behavioral shift was most prominent in the spring, when natural foods are scarce, and fall, when energetic demands are high. We also observed a high degree of inter-individual variability among our sample of bears. Bears with a higher density of houses in their home ranges (~ 75 houses/km2) displayed less avoidance of human development than more rural bears. Furthermore, bear movement models had different explanatory variables, with preference or avoidance of a variable being dependent on the individual bear. To account for this individuality in our predictive surfaces, we projected the probability of movement for each season and time of day using a spatially weighted surface centered on each bear’s home range.

**Conclusions:**

We found that black bears in Massachusetts are operating in a landscape of fear and are altering their movement patterns to use developed areas when human activity is low. We also found seasonal and diel differences among individual bears in resource selection during movement. Accounting for these individual, seasonal, and diel differences when assessing movement for large mammals is especially important if predictive surfaces are to be used in identifying areas for conservation and management.

**Electronic supplementary material:**

The online version of this article (10.1186/s40462-019-0166-4) contains supplementary material, which is available to authorized users.

## Background

Due to their space requirements, large mammals often exist partially or wholly outside of protected areas where they are exposed to varying levels of human presence. With the continued growth, expansion, and spatial redistribution of human populations, large mammals will increasingly encounter humans and human development [1, 2]. Human development has been shown to fragment and decrease the amount of habitat [3], reduce movements [4], and lower rates of survival and fecundity [5] for large mammals. However, developed areas may also provide food, either in the form of non-natural foods like garbage, agricultural crops, and livestock [6], or a higher density of natural foods like forage, rodents, and deer [7]. Because large mammals, especially carnivores, living in and around developed areas have been shown to increase human-wildlife conflict [8], understanding behavior and movement of these species in areas of human development is crucial for crafting effective education and management plans to reduce human-wildlife interactions.

The American black bear (*Ursus americanus*) is an opportunistic omnivore that can exploit anthropogenic foods such as garbage, bird seed, fruit trees, and agricultural crops. In developed areas, these supplemental foods can sometimes be an additional source of nutrition, however, bears in these areas also have an increased likelihood of mortality from vehicle collisions and lethal removal as well as decreased survival rates for subadults and adult females [3, 9–11]. A risk-reward tradeoff exists for bears living in and near human development, suggesting bears may be operating in a ‘landscape of fear’ – a conceptual model originally developed for prey species where an individual’s perception and use of its environment is the result of a cost-benefit analysis of food and risk [12–14]. Under this framework, the risk-reward tradeoffs for black bears would not only be influenced by the built environment, but also the energetic state of an individual and the presence of conspecifics [13].

Movement patterns can express how black bears perceive the human environment and the risk-reward trade-off [15, 16]. Previous studies have found black bears in relatively undisturbed areas move more during diurnal and crepuscular time periods than at night [9, 17], while black bears in areas of human development have been shown to shift movement activity to nocturnal periods [9, 18]. Johnson et al. [19] and Baruch-Mordo et al. [20] found black bears increase use of human development and are more active at night in years when natural food is scarce. Seasonally, bears have used human development more in spring, when natural food availability is typically low [19], and in fall when bears are in a state of hyperphagia [21]. These findings suggest black bears perceive human areas as risky, but will utilize these areas in times of low food availability to improve their energetic state. However, a black bear study in Pennsylvania, New Jersey, and West Virginia found no difference in response to human development between urban and rural black bears and concluded that uniform management actions could be applied across the entire bear population [22].

Clearly, there are still nuances in the movement patterns of black bears that can shed light on their perception of the risk-reward tradeoff in developed areas. The aforementioned studies have not examined movement in relation to different landscape features at different times of day, nor have they modeled movement with multi-scale models, which have been shown to be more appropriate than single scale models for modeling movement and habitat use [23, 24]. Furthermore, these studies did not predict movement in response to human development in a spatially-explicit manner that preserves the individuality of each bear and its presumed acclimation to human development [21].

Black bears have been observed to be highly individualistic in their response to landscape features [25]. Some of these individual responses may be the result of different genotypic or phenotypic expressions (e.g., boldness, curiosity, spatial learning) [26, 27], or sex, age, or reproductive status [21, 25, 28–30]. Other differences may be the result of acclimation to various landscape features in their home ranges [21]. Many studies account for individual variability in population-wide models by using random slopes and intercepts for individuals. Even with this approach, population models have been shown to mask individual differences, lead to weak or inconclusive responses to landscape features, and result in poor predictive results [25, 31]. Furthermore, spatial predictions have been solely based on population-level models despite spatially inhomogeneous responses across the landscape [32]. For example, bears in more rural areas may have a stronger negative response to roads and residential areas than bears in more developed environments. Modeling bears separately and then combining individual predictive surfaces into a single surface that preserves these spatial differences helps to avoid weak predictions and spatial biases [32].

We examine daily and seasonal black bear movement and response to human development in the Commonwealth of Massachusetts, USA. Massachusetts is the third most densely populated state in the nation [33] and has a growing black bear population that is expanding eastward from more rural parts of the state towards the greater Boston metropolitan area [34]. Our objectives were to determine how seasonal food resources and human development affected bear movement patterns and to spatially predict the probability of bear movement across the state. We first examined overall daily movement patterns as well as daily movement patterns in response to environmental features across seasons. We assumed that preference for moving through areas of human development at times other than peak movement times was evidence of bears altering their behavior to avoid risky situations. Second, we examined resource selection during movement by running a multi-scale step selection function (SSF) for each individual bear for each season during the day and at night. As part of this analysis we were interested in determining if bears with more human development in their home ranges would be more acclimated to human development or instead show higher avoidance of human development. Third, we predicted the probability of bear movement across the state for each season and diel period by spatially integrating the predicted surfaces for each individual weighted by the distance from each individual’s home range. The results from our analyses can be used to better understand bear perception and use of human development, project movement into areas of the state where bears are expanding, and provide valuable information for bear management and mitigation of human-wildlife conflicts.

## Methods

### Study area

**Fig. 1:**
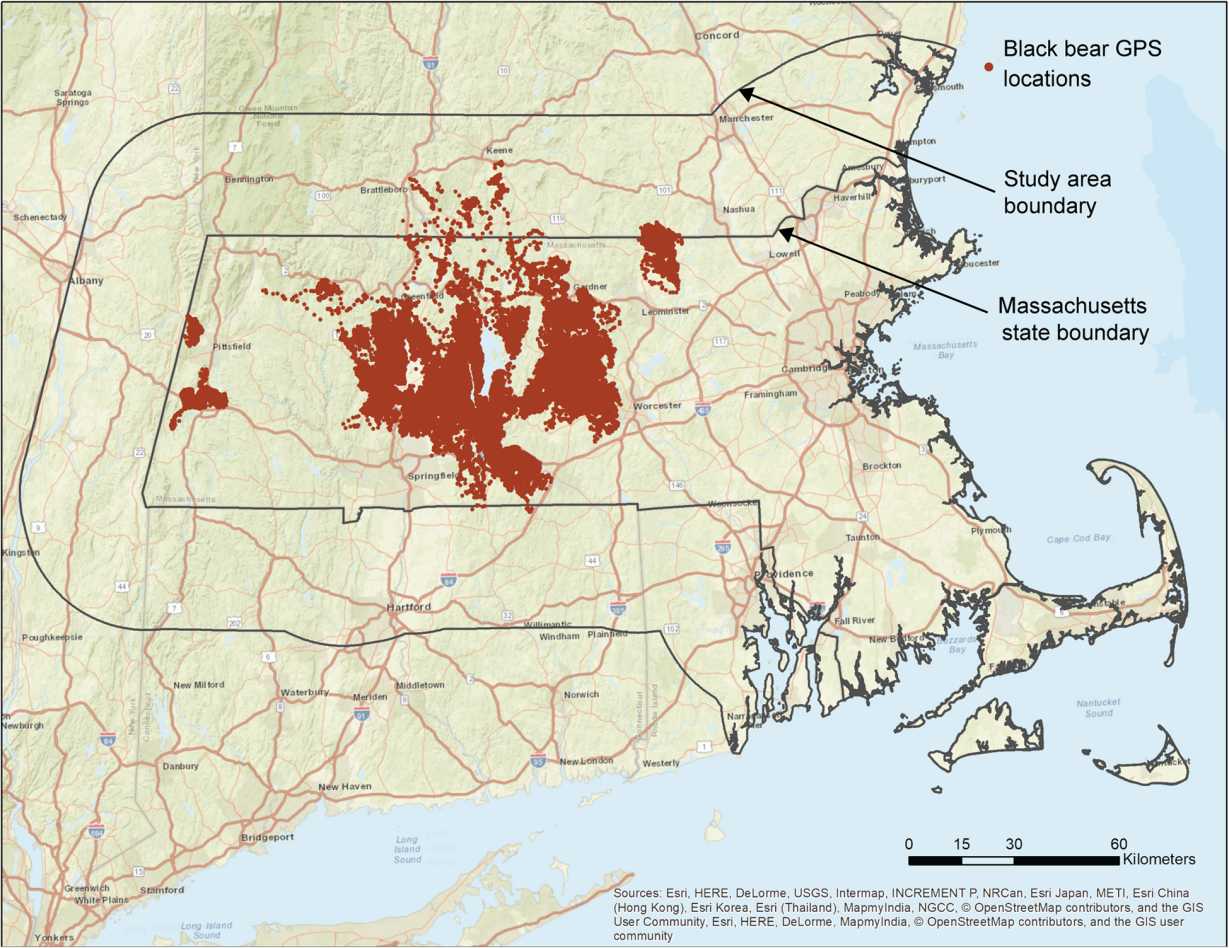
Location of black bear GPS points from bears collared with a 15 or 45-min fix interval

The study area was in the Commonwealth of Massachusetts, USA. Bears were collared and tracked in the central and western parts of the state (Fig. 1). To account for bears that moved beyond state borders, we buffered Massachusetts by 35 km to identify our greater study area. Spatial predictions were performed across the greater study area, but then were clipped to the state boundary.

### Black bear data

Female bears were captured between 2009 and 2017 and fitted with either a Telonics Gen 3 or Gen 4 GPS collar (Any use of trade, firm, or product names is for descriptive purposes only and does not imply endorsement by the U.S. Government). Most bear collars between 2012 and 2017 were programmed to acquire a fix every 15 (*n* = 7) or 45 (*n* = 69) minutes. These bears were used in the movement analyses. Bears with longer fix intervals (*n* = 20) were used as hold-out data to assess predictive performance of the movement models. GPS collar data for each bear (ID, fix interval, date range, etc.) are provided in Additional file 1: Table S1.

Bears were captured via barrel traps, free-range using dart projector, and during winter den checks. Bears were immobilized using 191 mg/ml Telazol ^R^ (Tiletamine HCl and Zolazepam HCl) at a dosage of 7 mg/kg in winter, and 229 mg/ml at a dosage of 5 mg/kg in summer, administered by a syringe on the end of a jab pole or via a tranquilizer dart. All capture, immobilization, and handling procedures were performed in accordance with University of Massachusetts, Amherst IACUC Protocols # 2011–0074, 2014–0074, and 2017–0066.

GPS fixes were examined for positional errors and the following categories of fixes were removed from the data set: (1) fixes that were unresolved, (2) fixes with a PDOP > 5 and classified as resolved QFP (uncertain) or 2D, (3) fixes with a PDOP > 20 and classified as resolved QFP (certain) or 3D. This filtering was done to minimize locational error [35] and resulted in a mean loss of 3.93% (SD = 2.67%) of the data points.

Den entry and den emergence were identified by visually examining the GPS points for each bear and identifying when movement stopped in the fall and began in the spring. Only points outside of the denning period were used in the analyses.

### Environmental variables

We selected a suite of landscape variables that may affect black bear movement (Table 1). Many variables were available across the entire study area. However, some variables were mapped more accurately in Massachusetts by the state Bureau of Geographic Information. Since most of our GPS locations were in Massachusetts, we combined the more accurate Massachusetts layers with region-wide layers by cross-walking categories from both data sets into a single layer. All raster variables were available at a 30 m pixel resolution. Vector data were rasterized to match this resolution. Though some small land use changes have occurred between the time the GIS data were collected and when the bear GPS data were obtained, land cover types are relatively stable in bear range in Massachusetts and no sweeping changes occurred over this period [37].

**Table 1:** Environmental variables used in the black bear movement analyses

Variable	Massachusetts source	Buffer area source (if different from Massachusetts source)
*Topographic*		
Ruggedness	derived from National Elevation Dataset	
Slope	derived from National Elevation Dataset	
*Development*		
All Roads	Designing Sustainable Landscapes	
Primary Roads	Designing Sustainable Landscapes	
Secondary & Tertiary Roads	Designing Sustainable Landscapes	
Primary, Secondary, & Tertiary Roads	Designing Sustainable Landscapes	
Open space	Massachusetts GIS Land Use 2005 layer (cemetery, golf course, recreation areas)	Designing Sustainable Landscapes (developed open space)
Commercial / Industrial	Massachusetts GIS Land Use 2005 layer (Commercial / Industrial / Junkyard / Urban Public, Institutional / waste disposal)	Designing Sustainable Landscapes (developed high intensity)
High density residential	Massachusetts GIS Land Use 2005 layer (Housing on < 1/4 acre lots)	Designing Sustainable Landscapes (developed high intensity)
Medium density residential	Massachusetts GIS Land Use 2005 layer (Housing on 1/4–1/2 acre lots)	Designing Sustainable Landscapes (developed medium intensity)
Low density residential	Massachusetts GIS Land Use 2005 layer (Housing on 1/2–1 acre lots)	Designing Sustainable Landscapes (developed low intensity)
Very low density residential	Massachusetts GIS Land Use 2005 layer (Housing on > 1 acre lots)	Designing Sustainable Landscapes (developed low intensity)
Percent impervious surface	Designing Sustainable Landscapes	
Agriculture	Massachusetts GIS Land Use 2005 layer (pasture, cropland, orchard, open land)	Designing Sustainable Landscapes (pasture)
Powerline corridors	Massachusetts GIS Land Use 2005 layer	Designing Sustainable Landscapes (developed open space)
*Water and wetlands*		
Open water	Massachusetts GIS Land Use 2005 layer	Designing Sustainable Landscapes
Emergent wetland	USGS National Wetlands Inventory	
Forested wetland	USGS National Wetlands Inventory	
*Vegetation*		
Coniferous forest	Pasquarella et al. [36]; Pasquarella et al. (*in prep*)	
Deciduous forest	Pasquarella et al. [36]; Pasquarella et al. (*in prep*)	
Mixed forest	Pasquarella et al. [36]; Pasquarella et al. (*in prep*)	
*Landscape Configuration Metrics*		
Similarity	Designing Sustainable Landscapes	

### Seasons

Ecologically-based seasons were identified with the clustering technique presented by Basille et al. [38]. This approach incorporates not only movement behavior, but also land cover attributes to account for changes in behavior based on food availability. We identified seasons using only data from bears with a 45-min fix interval (*n* = 69) so that speeds and turning angles were consistent. Data cleaning and missed GPS collar fixes resulted in the time between some locations being longer than the original collar schedule, therefore, we further subset the data so that only consecutive fixes were used. We then calculated movement speed and turning angle among consecutive points for each bear and assessed whether each point was located in coniferous forest, deciduous forest, forested wetland, or agricultural areas. We also determined the value of percent impervious surface at each point. At the recommendation of Basille et al. [38], we then smoothed the movement and habitat variables for each bear-year with a 5-day moving window and range standardized the data. We used k-means clustering and selected the number of clusters with the gap statistic [38]. This approach resulted in three seasons: spring (den emergence to June 14th), summer (June 15th to August 9th), and fall (August 10th to den entry). For the seasonal analyses presented below, only bears that had a full season’s worth of data were used.

### Movement steps

Because our interest was in longer movements across the landscape, as opposed to short distance or foraging movements, we identified movement behavior from the step length and turning angle distributions of each bear by using hidden Markov models with the R package [39] *moveHMM* [40]. We ran a three-state hidden Markov model which characterized behavior as (1) an encamped state with short steps and wider turning angles, (2) a foraging state with slightly longer steps and smaller turning angles, and (3) a movement state with long step lengths and directed movement (more details and an example are provided in Additional file 2: Figure S2). We used only movement steps for all subsequent analyses.

### Statistical analyses

#### Daily movement and habitat selection

Following VanCleave et al. [41], for the bears with a 45-min fix interval, we calculated the bootstrapped mean and confidence intervals of step lengths over each 45-min period throughout the day for each season. We also calculated the third-order selection ratio (sensu Johnson [42]) for each bear for each categorical predictor variable for each 45-min period. The selection ratios were estimated for each individual by first calculating the total proportion of each categorical variable within its seasonal 95% kernel density home range (Additional file 3). We then calculated the mean proportion of each step that was in each predictor variable for each 45-min time period and divided this by the proportion of that variable in an individual’s seasonal home range. The bootstrapped mean and confidence intervals for each 45-min time period were calculated across bears.

#### Multi-scale step selection functions (SSF)

Due to differences observed in bear temporal movement and habitat selection, we ran two SSFs for each season, one with daytime points and one with nighttime points. Day/night periods for each bear were identified using daily sunrise/sunset times for Amherst, Massachusetts from the Astronomical Applications Department of the U.S. Navy Observatory. We also ran a single SSF with all the bear data across seasons and diel periods. Bears with the 15-min or 45-min fix interval were used for this analysis.

Following Zeller et al. [23], the mean value (for continuous variables) or proportion of the area (for categorical variables) was extracted within a 30 m uniform buffer around each step. This was the ‘used’ data for the SSFs. We examined multiple scales for representing the ‘available’ data. Scales were determined by estimating the mean distance moved over the following time periods: 45 min, 1.5 h, 2.25 h, 3 h, 4.5 h, and 6 h. The mean movement distances were 360 m, 650 m, 837 m, 1007 m, 1288 m, and 1523 m respectively. The scales were used as the standard deviation for a Gaussian kernel placed over each step. The mean value or proportion of each variable was calculated within the Gaussian weighted kernel around each step at each scale and were used as the ‘available’ data. Used and available data were z-score standardized prior to modeling.

We modeled each bear independently to preserve individual differences and predict the relative probability of movement in a spatially unbiased manner (see Predictive Surfaces below) [32]. We paired each used step with its corresponding available step at a scale and ran paired logistic regressions with the clogit function from the R package coxme [43]. For each bear, we used a two-step approach to generate the SSF [24]. We first ran univariate models for each variable at each scale. We identified the scale with the lowest AICc value as the characteristic scale of selection for that variable. We then assessed pairwise correlations between the variables at their characteristic scales. If pairwise correlations were | > 0.7|, we selected the variable from each pair with the lower AICc value for inclusion in the multiple regression models. We ran a forward and backward stepwise model selection and identified the final model for each bear as the model with the lowest AICc value.

#### Home range housing density effects on selection

To determine if bears living in more developed areas were more acclimated to human development, we modeled the standardized regression coefficient for percent impervious surface as a function of home range housing density (HRHD) [21]. We calculated HRHD for each bear’s annual home range as well as each bear’s seasonal home range. For each season, we explored a linear relationship and a logistic relationship. We also ran an intercept only model. Competing models were ranked with AICc values. We assumed that if the highest ranked model included HRHD as a variable and that if the slope of this model was positive, then bears were acclimating to residential areas within their home ranges.

### Predictive surfaces

Because we observed significant results from our HRHD analysis, we concluded that bears in more developed areas were responding to landscape features differently than bears in undeveloped areas. To maintain this individual variability in our predictive surfaces we did not average the model coefficients across bears (which does not maintain individual variability in the predictive surface), but instead used the approach presented in Osipova et al. [32], which spatially weights predictions for each individual based on their home range centroid. By projecting each bear model separately across the study area, but combining them through a spatial weighting by distance from home range centroid, a single surface is obtained. To generate this predictive surface, we used the following procedure. For each bear year, we predicted the relative probability of movement across Massachusetts using the exponentiated form of the relative predicted resource selection probabilities as recommended by Johnson et al. [44]: $\hat{w}\ (x)=\exp \Big({\beta}_1{x}_1+{\beta}_2{x}_2+{\beta}_3{x}_3+$ … + *β*_*p*_*x*_*p*_). Before combining bear years into a single surface, we weighted each bear’s surface so that areas close to each bear’s home range had a higher weight than areas further away. We implemented this by (1) estimating a kernel density home range for each bear for each season (Additional file 3), (2) identifying the home range centroid, (3) generating a distance from home range centroid surface, and (4) taking the inverse of the distance surface so that higher values were closer to the home range centroid [32]. This was our weighted surface. Each pixel on the landscape was normalized so that all the home range weighted surfaces summed to one. We then multiplied each relative predictive surface for each bear by the home range weighted surface and summed them to obtain a final predicted probability surface across the state for all seasons and for each season/diel period.

We evaluated the performance of the predictive surfaces using the hold-out data set of 20 bears and calculating the Boyce Index [45, 46], which compares the predicted values at the hold-out points with the expected values across the study area. The Boyce Index has values ranging from 0 to 1 with values closer to 1 indicating better predictive ability. We compared the predictive surfaces with each other by calculating the Pearson’s correlation coefficient and by the pixel by pixel differences between the surfaces.

## Results

### Daily movement and habitat selection

**Fig. 2:**
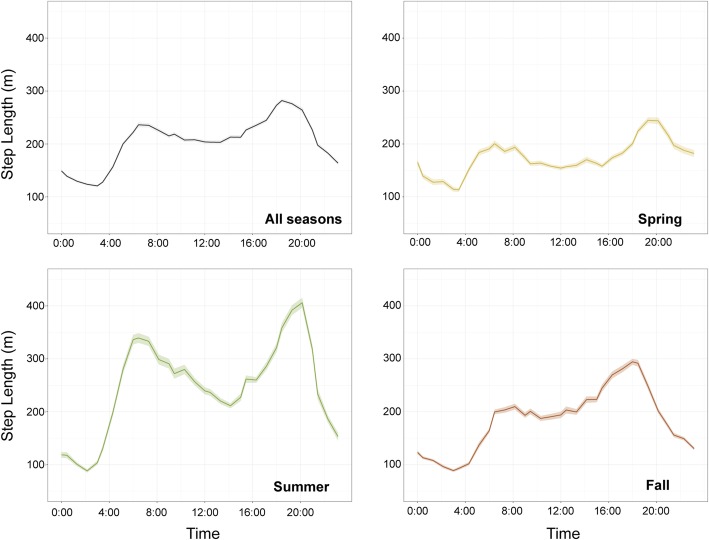
Mean step length and confidence intervals for each 45-min period throughout the day

Across seasons, bears moved an average of 200 m every 45-min during daylight hours (Fig. 2). Movement steps were longer during crepuscular periods with the longest steps observed during the evening hours from approximately 16:00–21:00 h. The minimum movement lengths occurred from approximately 02:00–03:00 h and remained low during most nighttime hours. Seasonally, movement activity maintained the same general patterns, but bear steps were shorter during the spring than during other times of year and were longer during summer than during other times of year (Fig. 2).

**Fig. 3:**
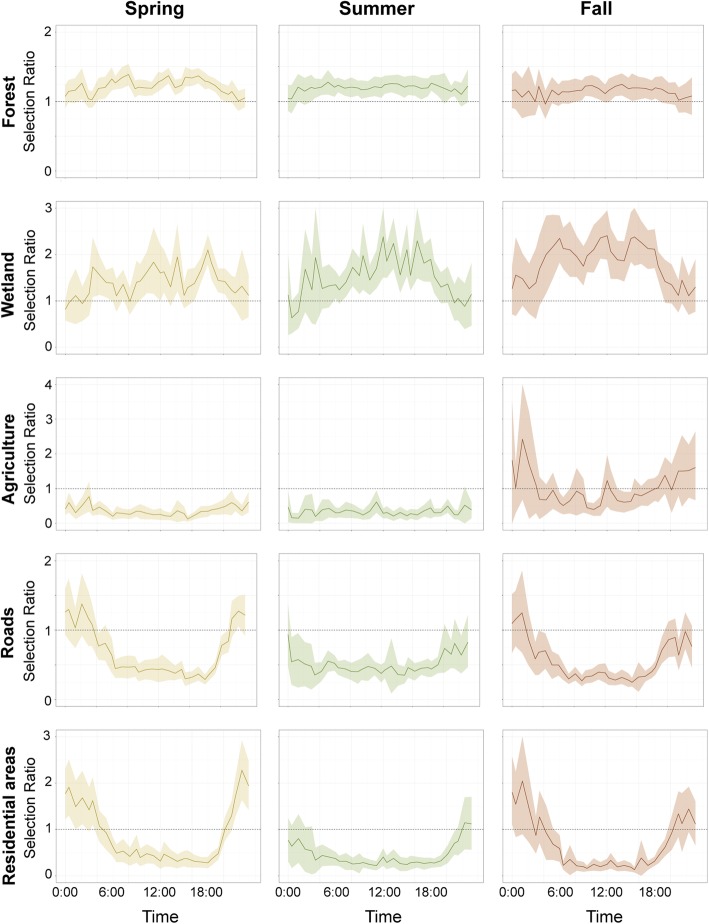
Selection ratios for forest, wetland, agriculture, roads, and residential areas for each 45-min window. All forest, wetland, and residential types were combined in these analyses

The selection ratio analysis indicated bears selected for forested areas across seasons and times of day and selected for wetlands strongly during daylight hours (Fig. 3). Bears avoided agricultural areas at all times of day in spring and summer, but bears selected for agriculture in the fall during the nighttime hours and showed variable selection in the fall during the daytime. Agriculture in our study area includes crops like corn, berries, apples, and a wide variety of vegetables bears may be utilizing. Bears had similar responses to roads and human development; bears selected for roads and residential areas at night during spring and fall, but strongly avoided these areas during the day. Bears mostly selected against roads and residential areas in the summer at all times of day with the exception of showing a slight tolerance for residential areas at night.

### Multi-scale step selection functions

The multi-scale SSF models for individual bears often contained different variables and different numbers of variables. The all-seasons model for each bear included 3 to 11 landscape variables with an average of 7.68 ± 1.84 variables per model. For the natural cover types, forested wetland was present in 80% of the models, and mixed and deciduous forest were both present in 73% of the models (Table 2). For the developed cover types, percent impervious surface was present in 61% of the models, agriculture was present in 55% of the models, and the other variables were included in the bear models less often. High and medium density residential variables were not selected for any final bear model, though low and very low density residential variables were present in some of the models. Similar results were observed for each season/diel period with forested wetland, mixed and deciduous forest, impervious surface, and agriculture being present in a high number of models (Table 2).

**Table 2:** Proportion of bear models containing each environmental variable. Because the step selection functions were optimized for each individual bear, not all bears had the same explanatory variables in their final models

	All seasons/diel periods	Spring day	Spring night	Summer day	Summer night	Fall day	Fall night
Percent impervious surface	0.61	0.29	0.14	0.55	0.33	0.35	0.30
All Roads	0.17	0.34	0.19	0.13	0.19	0.28	0.30
Secondary & Tertiary Roads	0.10	0.15	0.14	0.17	0.13	0.15	0.11
Primary, Secondary, & Tertiary Roads	0.11	0.16	0.14	0.13	0.19	0.21	0.15
Primary Roads	0.06	0.05	0.05	0.02	0	0.07	0
Very low density residential	0.14	0.24	0.38	0.21	0.19	0.12	0.26
Low density residential	0.18	0.11	0.43	0.19	0.14	0.12	0.22
Commercial / Industrial	0.23	0.21	0.29	0.17	0.10	0.09	0.07
Open space	0.14	0.05	0.24	0.06	0.05	0.05	0.11
Agriculture	0.55	0.47	0.48	0.43	0.43	0.40	0.48
Powerline corridors	0.30	0.05	0.14	0.32	0.14	0.19	0.22
Slope	0.48	0.34	0.29	0.30	0.24	0.37	0.26
Ruggedness	0.52	0.26	0.24	0.49	0.33	0.47	0.33
Similarity	0.48	0.53	0.43	0.34	0.38	0.56	0.48
Open water	0.26	0.21	0.48	0.15	0.14	0.14	0.19
Emergent wetland	0.44	0.50	0.48	0.43	0.24	0.23	0.30
Forested wetland	0.80	0.53	0.29	0.64	0.24	0.81	0.52
Coniferous forest	0.55	0.39	0.43	0.34	0.33	0.35	0.33
Mixed forest	0.66	0.47	0.38	0.44	0.57	0.53	0.44
Deciduous forest	0.73	0.58	0.33	0.57	0.38	0.67	0.70

Bears also responded to different landscape variable scales (Additional file 4: Figure S4). Across seasons and times of day, bears typically selected for finer scales of forested wetland, conifer forest, primary roads, and commercial/industrial lands and selected for coarser scales of emergent wetland, agriculture, low and very low density residential areas, imperviousness, secondary and tertiary roads, deciduous and mixed forest, slope, and ruggedness. However, the scale of selection was not consistent across bears and individual bears selected varying scales for all landscape variables (Additional file 4: Figure S4).

**Fig. 4:**
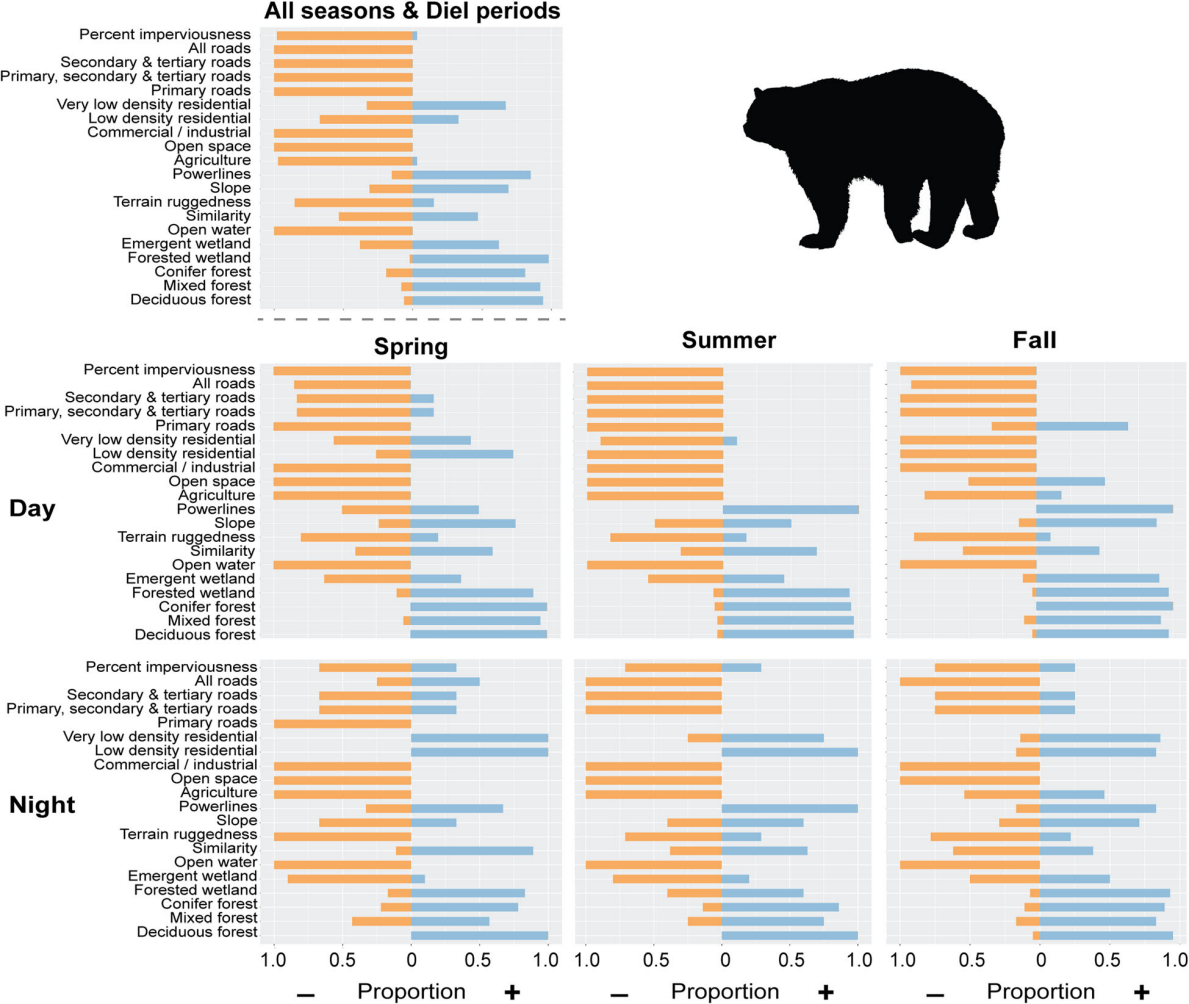
Proportion of bear movement models with a positive or negative regression coefficient for a variable. Blue bars indicate the proportion of models with positive coefficients and orange bars indicate the proportion of models with negative coefficients. Because the step selection functions were optimized for each individual bear, not all bears had the same explanatory variables in their final models

The response to environmental variables differed across the bear population. Figure 4 shows the proportion of the final bear models having a positive or negative coefficient for each variable. Generally, across seasons and diel periods, bears preferred forested cover types and wetlands and avoided agriculture, open water, open space, roads, and high values of percent impervious surface (Fig. 4). However, responses to these landscape features were not consistent and changed with season and time of day. For each season, a higher proportion of bear models showed selection for percent impervious surfaces, roads, and other residential development at night than during the day. All of the models for spring night showed a selection for low and very low residential development. For spring and summer during both day and night, bear models all showed an avoidance of agriculture, but for fall day, about 25% of models showed a preference for agriculture and for fall night, almost 50% of bear models showed a preference for agriculture.

### Home range housing density analysis

**Fig. 5:**
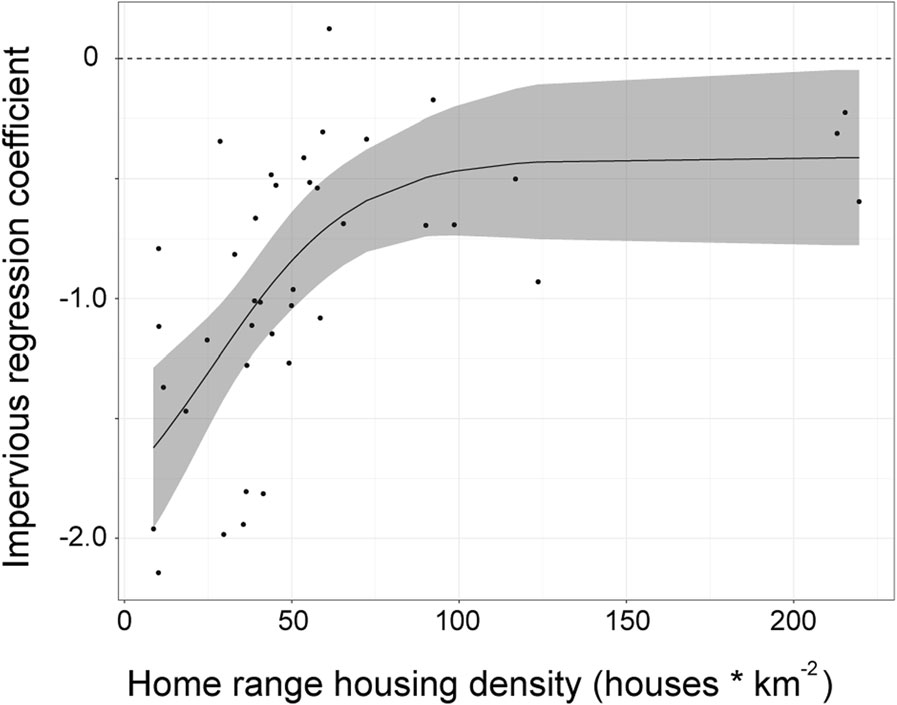
Logistic relationship between the home range housing density for each bear and the standardized regression coefficient for percent impervious surface from the step selection function models

Bears with a higher HRHD showed weaker avoidance of human development compared with more rural bears (Fig. 5). The logistic model was the best fitting model and was significant. AICc values of the logistic model, the linear model and the intercept only model were − 10.5, − 3.8, and 5.3 respectively. The threshold of this curve was observed around a housing density of 75–100 houses/km^2^. Because the percent impervious surface variable was not included in all the bear models, when we parsed the data into seasons, we did not have enough data to fit the HRHD models to each season.

### Probability of movement surfaces

**Fig. 6:**
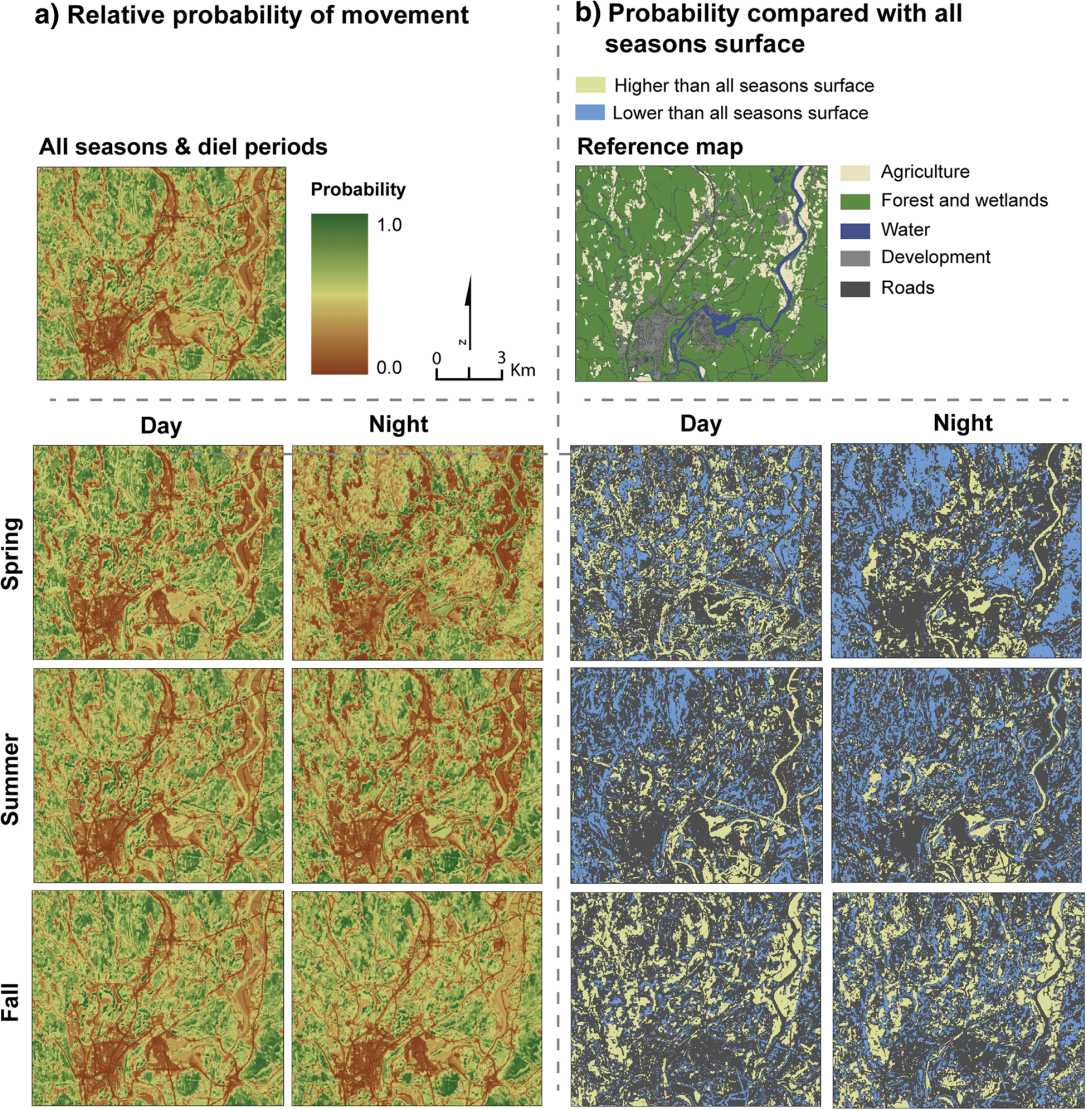
(**a**) Spatially weighted relative probability of movement surfaces for each season/diel period and all seasons/diel periods. A small part of the Commonwealth of Massachusetts is shown. See reference map for major land cover types in this region. (**b**) Differences in the relative predicted probability of movement surfaces. Each seasonal/diel period surface was subtracted from the all seasons/diel periods surface. A positive difference indicates that the pixel in the seasonal/diel period surface had a lower probability of movement than that same pixel in the all seasons surface. A negative difference indicates that the pixel in the seasonal/diel period surface had a higher probability of movement than the all seasons surface. The top 20% of positive differences (blue) and top 20% of negative differences (yellow) are shown.

Differences in habitat selection among the season/diel models were reflected in the probability of movement surfaces (Fig. 6a; statewide surfaces are provided in Additional file 5: Figure S5). The correlations between the all seasons/diel periods probability surface and the other surfaces were all relatively high (spring day = 0.97, spring night = 0.62, summer day = 0.98, summer night = 0.86, fall day = 0.98, fall night = 0.94). However, differences between the surfaces on a pixel by pixel basis could be quite pronounced. Figure 6b shows the top 20^th^ quantiles of positive and negative differences between the all seasons/diel periods surface and the other surfaces. Positive differences indicate that a higher probability of selection was observed for the pixels in that season/diel period than in the all seasons model. Negative differences indicate that a lower probability of selection was observed for that season/diel period. Positive differences in relative probability values ranged from 0.05 to 0.47 for daytime surfaces and 0.09 to 0.54 for nighttime surfaces. Negative differences in relative probability values ranged from − 0.05 to − 0.45 for daytime surfaces and − 0.12 to − 0.92 for nighttime surfaces.

All the predictive surfaces had relatively high predictive performance. The Boyce Index Spearman rank correlation coefficient values were 0.98, 0.95, 0.72, 0.98, 0.83, 0.98, and 0.93 for the all seasons, spring day, spring night, summer day, summer night, fall day, and fall night surfaces respectively.

## Discussion

In keeping with the ‘landscape of fear’ model [12, 14], our study on bear movement patterns indicates that black bears in Massachusetts mitigate the risk of being in areas of human development by altering their behavior to exploit these areas when human activity is low. Nocturnal utilization of developed areas was strongest in the spring, when natural foods are more limited, and in the fall, when bears are in a state of hyperphagia. Our study also found that bears with higher amounts of human development in their home ranges did not avoid human areas as strongly as more rural bears, indicating bears are acclimating to living in developed areas. Given the differences among bears living in rural and more developed areas, our spatial predictions of black bear movement preserved the individual nature of bears exposed to different landscape features.

Black bear movement patterns were typical of black bears in wildland areas with most movement occurring during the day, with peaks during morning and evening hours [9, 17]. However, examining daily selection for different landscape features showed that when bears did move at night, they displayed a preference for roads and residential areas, especially in the spring and fall. This is a shift from normal activity patterns and suggests behavioral plasticity in response to human development. Similar nocturnal shifts have been observed in other black bear studies in developed areas [9, 18–20]. Black bears may be shifting their use of developed areas to times when human activity is low, indicating black bears perceive human activity and associated noise and traffic as risky and may be responding to these environmental cues more than human infrastructure on the landscape [21, 47]. These shifts are not only occurring in black bears, but have also been documented in other species. In a meta-analysis on 62 species, Gaynor et al. [48] found that large mammals significantly increased nocturnal activity in response to all types of human presence.

Limitations of natural food sources as well as the energetic state of individual bears may drive when bears are willing to utilize riskier human dominated areas. We found black bear use of developed areas at night was strongest in the spring and fall, indicating these behavioral shifts can be dynamic throughout the year and may be tied to caloric requirements. In our study area, the availability of natural foods in the spring is lower than other times of year, and in the fall, bears are in a state of hyperphagia and have increased metabolic requirements. In the summer, though movement rates of bears are higher than the other two seasons, this nocturnal shift is not as prominent, suggesting greater availability of natural food sources in less risky environments and that bears weigh risks with energetic demands. Baruch-Mordo et al. [20] and Johnson et al. [19] found that the shift to increased activity in developed areas was coincident with poor food years. We did not measure natural food availability directly, so were unable to study whether bears in Massachusetts exhibited higher use of human development in poor food years. Furthermore, our study only included female bears, but Johnson et al. [19] found use of human development was also dependent on sex and age, and Evans et al. [21] found use of development was a function of reproductive status, lending further evidence that use of human development is temporally dynamic. Expanding this research to include male bears as well as bears in different age classes and reproductive states would allow for a more nuanced picture of black bear response to human development in Massachusetts. Additionally, this research could be enhanced by using time of day as a continuous variable in the SSF models instead of using a binary classification of day and night.

The unit of inference for our SSF analysis was the step between consecutive GPS collar fixes. Previous studies have found that as the time between fixes increases, the paths of individuals become less tortuous and shorter in length [49], and biases may be introduced into SSFs at fix rates of one hour or more [23]. Our steps were at 15 and 45-min intervals and though this period may not be short enough to completely capture the more tortuous bear movements, we are confident in our inferences. The results are biologically meaningful and echo previous research on bear habitat use and movement.

Bears with a higher housing density in their home ranges had a significantly weaker avoidance of impervious surfaces, indicating bears were acclimating to human development. This relationship was best expressed as a threshold, where weaker avoidance was observed at home range housing densities above approximately 75 houses/km^2^. Evans et al. [21] also found a significant relationship between home range housing density and development and that bears showed a preference for development when their home range housing density was over 66 houses/km^2^. In our study, bears did not show preference, but instead, weaker avoidance with home range housing density. Differences among these studies are likely due to the use of different measures of development. Evans et al. [21] used housing density whereas we used percent impervious surface, which is not restricted to buildings, but includes roads and other surfaces associated with development. Furthermore, our sample of bears included bears in areas that were more highly developed than those in the Evans et al. [21] study.

Bears in Massachusetts showed a high degree of inter-individual variability. Not only were bears in human dominated areas more acclimated to development than other bears, but also movement of individual bears was driven by different landscape features. Bear movement models had different numbers and types of variables and preference or avoidance of a variable was dependent on the individual bear. In general, bear movement was facilitated by forest and forested wetland and impeded by roads and developed areas, but this was not entirely consistent across the population especially when seasonality and time of day was considered. Lesmerises and St-Laurent [25] observed individuality in black bears and found opposite selection coefficients that were linked to individual characteristics. They also found that when all bears were included in a population-level model, these opposite responses cancelled each other out and resulted in weak selection responses. For an omnivorous, generalist species such as the black bear, that is capable of behavioral plasticity depending on its environment, ignoring individual variability can result in inconclusive or misleading results [25].

Accounting for individual variability by modeling each individual separately provides important insights for understanding wildlife habitat use and movement. However, applying individual models in a spatially explicit manner to project habitat use or movement across a study area is impractical due to the sheer number of individual surfaces this would produce. This has prevented the use of individual models in spatially projecting resource use and movement. Osipova et al. [32] provides a nice solution to this problem with an approach that maintains the individuality of each bear relative to its location in a study area. By projecting each bear model separately across the study area, but combining them through a spatial weighting by distance from home range centroid, a single surface is obtained. Osipova et al. [32] demonstrated that this spatial weighting approach outperformed the surface derived from a population level model where the regression coefficients were averaged. We implemented the Osipova et al. [32] approach and produced predictive movement surfaces spatially weighted by each individual bear. The high Boyce Index values indicate high predictive performance of these models. Because the individual-based spatially weighted approach is still a relatively new method, more research is needed to determine how many individuals and over what spatial configuration is ‘enough’ to result in meaningful predictions. Additionally, Signer et al. [50], recommended a simulation-based approach for generating the predictive surfaces from SSF models as they found it to perform better than the traditional approach of multiplying the coefficients with the geospatial variables. Some combination of the individual-based spatially weighted approach proposed by Osipova et al. [32], and the simulation-based approach proposed by Signer et al. [50] may further improve predictive surfaces generated from SSFs, however more research on this topic is needed.

Accounting for time of day and season was also important in our predictive surfaces. Bear movement may be interpreted differently depending on the time of day and season and it is important to account for these differences when assessing movement across the landscape, especially if predictive surfaces are to be used in identifying areas of connectivity, conservation corridors, and road crossing locations.

## Conclusions

Understanding the movement of large mammals in and around areas of human development is crucial for developing successful management plans and mitigation approaches for reducing human wildlife conflict. We analyzed black bear movement in the third most densely populated state in the U.S. and observed behavioral plasticity and acclimation to human development. We also observed inter-individual differences and suggest maintaining these inter-individual differences when spatially predicting movement across a landscape. We were unable to assess whether use of developed areas conferred positive or negative fitness benefits, but this is a question for future research. Our results offer insights into the landscape of fear for black bears and what drives their spatial and temporal use of human dominated areas and may be used by managers across highly developed landscapes, such as the state of Massachusetts. Our predictive surfaces will also help identify how and where bears are expanding as they move closer to the Boston metropolitan area.

## Additional files

Additional file 1: **Table S1.** Bear-specific GPS collar data. (DOCX 24 kb)

Additional file 2: **Figure S2.** Example results from a three-state hidden Markov model for a single bear. (DOCX 441 kb)

Additional file 3: Kernel Density Home Range Estimation (DOCX 14 kb)

Additional file 4: **Figure S4**: Spatial scales of selection for black bear step selection functions. (DOCX 655 kb)

Additional file 5: **Figure S5:** Relative probability of black bear movement across the state of Massachusetts (DOCX 1689 kb)

## Data Availability

The data are available upon request from the authors.

## Abbreviations

HRHD: Home range housing density
SSF: Step selection function
