# Supplementary material for: Black bears alter movements in response to anthropogenic features with time of day and season
Source: Mov Ecol. 2019 Jul 11;7:19. doi: 10.1186/s40462-019-0166-4 (PMC6621962; doi:10.1186/s40462-019-0166-4)
Supplement: Supplementary file 1 — Table S1. Bear-specific GPS collar data. (DOCX 24 kb) [file 40462_2019_166_MOESM1_ESM.docx]

**Additional File 1**

**Table S1.** Bear-specific GPS collar data including individual ID, year, fix interval, start and end dates of collar deployment, number of fixes after data filtering, and reproductive status.

| **ID** | **Year** | **Fix interval (minutes)** | **Full date range of data** | | **Number of fixes cleaned and outside denning period** | **Reproductive status** |
| --- | --- | --- | --- | --- | --- | --- |
|  |  |  | **start** | **end** |  |  |
| 280 | 2009 | 90-150 | 2009.03.10 | 2009.09.15 | 1272 | newborn |
| 309 | 2009 | 90 - 150 | 2009.02.10 | 2010.01.12 | 1780 | none |
| 269 | 2009 | 90-150 | 2009.03.05 | 2010.02.28 | 1773 | newborn |
| 318 | 2010 | 90-150 | 2010.03.05 | 2011.01.06 | 1538 | newborn |
| 298 | 2010 | 90 - 150 | 2010.02.19 | 2010.11.23 | 1751 | yearling |
| 298 | 2011 | 85 | 2011.03.04 | 2012.02.05 | 2349 | newborn |
| 234 | 2011 | 85 | 2011.02.23 | 2011.12.29 | 1963 | newborn |
| 310 | 2011 | 85 | 2011.03.03 | 2012.01.05 | 1951 | yearling |
| 258 | 2011 | 85 | 2011.03.08 | 2012.03.07 | 257 | newborn |
| 345 | 2011 | 90 - 150 | 2010.05.11 | 2010.11.23 | 937 | yearling |
| 253 | 2011 | 85 | 2011.03.14 | 2012.01.05 | 2028 | yearling |
| 318 | 2011 | 85 | 2011.02.18 | 2011.09.18 | 1423 | yearling |
| 323 | 2011 | 85 | 2011.03.15 | 2012.03.15 | 3379 | newborn |
| 234 | 2012 | 45 | 2012.02.22 | 2013.03.11 | 3521 | yearling |
| 310 | 2012 | 45 | 2012.03.28 | 2013.02.11 | 4039 | newborn |
| 258 | 2012 | 45 | 2012.03.14 | 2013.02.08 | 3668 | yearling |
| 345 | 2012 | 85 | 2011.03.09 | 2011.12.13 | 1717 | newborn |
| 349 | 2012 | 75 | 2012.03.07 | 2012.12.24 | 2112 | newborn |
| 253 | 2012 | 45 | 2012.03.09 | 2013.02.07 | 4186 | newborn |
| 357 | 2012 | 75 | 2012.03.09 | 2012.10.30 | 2166 | newborn |
| 355 | 2012 | 45 | 2012.09.13 | 2013.02.28 | 393 | none |
| 376 | 2012 | 85 | 2012.06.05 | 2013.02.28 | 950 | yearling |
| 323 | 2012 | 45 | 2012.03.21 | 2013.03.01 | 3770 | yearling |
| 258 | 2013 | 45 | 2013.03.17 | 2014.02.27 | 3277 | newborn |
| 298 | 2013 | 45 | 2013.04.02 | 2014.03.25 | 6503 | newborn |
| 388 | 2013 | 45 | 2013.02.21 | 2013.09.11 | 4262 | yearling |
| 376 | 2013 | 85 | 2013.03.02 | 2014.05.06 | 2035 | newborn |
| 393 | 2013 | 45 | 2013.05.09 | 2014.02.24 | 3624 | newborn |
| 310 | 2013 | 45 | 2013.02.15 | 2014.03.07 | 4743 | yearling |
| 395 | 2013 | 45 | 2013.05.21 | 2014.01.19 | 3726 | newborn |
| 349 | 2013 | 75 | 2013.03.10 | 2014.03.18 | 2233 | yearling |
| **ID** | **Year** | **Fix interval (minutes)** | **Full date range of data** | **Number of fixes cleaned and outside denning period** | **Reproductive status** | **ID** |
| 391 | 2013 | 45 | 2013.05.02 | 2014.01.17 | 2786 | none |
| 356 | 2013 | 45 | 2013.05.01 | 2014.03.17 | 4410 | none |
| 253 | 2013 | 45 | 2013.02.23 | 2014.03.06 | 5845 | yearling |
| 370 | 2013 | 45 | 2013.03.06 | 2014.02.28 | 4475 | newborn |
| 323 | 2013 | 45 | 2013.03.02 | 2013.09.09 | 2307 | none |
| 355 | 2013 | 45 | 2013.03.01 | 2014.03.10 | 3452 | newborn |
| 357 | 2013 | 75 | 2013.04.11 | 2013.11.09 | 1849 | unknown |
| 373 | 2013 | 45 | 2013.03.05 | 2014.03.14 | 2727 | none |
| 393 | 2014 | 45 | 2014.02.22 | 2015.03.02 | 4239 | yearling |
| 298 | 2014 | 45 | 2014.03.26 | 2014.06.17 | 1065 | yearling |
| 406 | 2014 | 45 | 2014.06.19 | 2015.02.12 | 2114 | unknown |
| 258 | 2014 | 45 | 2014.02.28 | 2015.02.17 | 3665 | yearling |
| 391 | 2014 | 45 | 2014.03.11 | 2015.02.10 | 3805 | none |
| 356 | 2014 | 45 | 2014.02.26 | 2015.01.08 | 5024 | newborn |
| 373 | 2014 | 45 | 2014.03.15 | 2014.11.01 | 3365 | none |
| 269 | 2014 | 45 | 2014.02.24 | 2015.03.02 | 3481 | yearling |
| 355 | 2014 | 45 | 2014.03.10 | 2015.01.28 | 4693 | newborn |
| 370 | 2014 | 45 | 2014.03.01 | 2015.01.01 | 3271 | yearling |
| 406 | 2015 | 45 | 2015.03.11 | 2016.01.05 | 4102 | newborn |
| 426 | 2015 | 45 | 2015.05.30 | 2016.01.04 | 3647 | newborn |
| 310 | 2015 | 45 | 2015.03.10 | 2015.05.19 | 837 | yearling |
| 393 | 2015 | 45 | 2015.03.10 | 2015.12.01 | 4043 | newborn |
| 391 | 2015 | 45 | 2015.03.02 | 2016.01.09 | 5003 | newborn |
| 395 | 2015 | 45 | 2015.03.05 | 2016.01.19 | 5153 | newborn |
| 403 | 2015 | 45 | 2015.02.19 | 2015.11.23 | 2973 | none |
| 356 | 2015 | 45 | 2015.03.06 | 2015.12.13 | 3318 | newborn |
| 356 | 2015 | 45 | 2015.12.21 | 2106.12.06 | 3287 | newborn |
| 404 | 2015 | 45 | 2015.02.12 | 2015.06.18 | 1526 | none |
| 432 | 2015 | 45 | 2015.07.15 | 2015.12.14 | 2594 | newborn |
| 428 | 2015 | 45 | 2015.05.01 | 2016.01.04 | 2590 | none |
| 425 | 2015 | 45 | 2015.05.30 | 2016.01.04 | 2110 | newborn |
| 373 | 2015 | 45 | 2015.03.16 | 2015.08.25 | 2632 | newborn |
| 269 | 2015 | 45 | 2015.03.13 | 2015.11.10 | 3176 | newborn |
| 355 | 2015 | 45 | 2015.01.29 | 2015.12.02 | 802 | yearling |
| 355 | 2016 | 15 | 2016.03.11 | 2017.01.23 | 16823 | newborn |
| **ID** | **Year** | **Fix interval (minutes)** | **Full date range of data** | **Number of fixes cleaned and outside denning period** | **Reproductive status** | **ID** |
| 436 | 2016 | 45 | 2016.03.22 | 2016.12.06 | 4492 | newborn |
| 425 | 2016 | 45 | 2016.03.09 | 2016.12.13 | 5025 | newborn |
| 428 | 2016 | 15 | 2016.01.04 | 2017.01.02 | 11102 | newborn |
| 432 | 2016 | 45 | 2016.02.16 | 2017.01.23 | 3752 | yearling |
| 424 | 2016 | 45 | 2016.03.11 | 2016.12.27 | 4320 | newborn |
| 403 | 2016 | 15 | 2016.02.29 | 2016.05.16 | 1488 | newborn |
| 395 | 2016 | 45 | 2016.01.19 | 2016.12.20 | 4515 | yearling |
| 391 | 2016 | 15 | 2016.03.03 | 2016.12.04 | 11272 | yearling |
| 406 | 2016 | 45 | 2016.01.05 | 2016.12.06 | 4185 | yearling |
| 426 | 2016 | 45 | 2016.02.01 | 2016.12.26 | 4883 | yearling |
| 309 | 2016 | 15 | 2016.05.06 | 2016.12.13 | 8508 | yearling |
| 253 | 2016 | 15 | 2016.03.04 | 2016.07.17 | 4737 | newborn |
| 370 | 2016 | 15 | 2016.05.24 | 2016.12.27 | 7014 | yearling |
| 433 | 2017 | 45 | 2017.02.03 | 2017.11.27 | 4211 | none |
| 460 | 2017 | 45 | 2017.05.11 | 2017.06.05 | 453 | newborn |
| 452 | 2017 | 45 | 2017.04.26 | 2017.05.23 | 289 | yearling |
| 426 | 2017 | 45 | 2017.03.09 | 2017.11.28 | 4814 | newborn |
| 450 | 2017 | 45 | 2017.02.23 | 2017.11.14 | 4024 | none |
| 476 | 2017 | 45 | 2017.07.06 | 2017.12.21 | 3092 | none |
| 309 | 2017 | 45 | 2017.03.10 | 2017.12.01 | 5052 | newborn |
| 470 | 2017 | 45 | 2017.06.16 | 2018.01.07 | 2410 | none |
| 424 | 2017 | 45 | 2017.02.24 | 2017.12.04 | 4415 | yearling |
| 472 | 2017 | 45 | 2017.06.20 | 2017.12.18 | 3186 | none |
| 432 | 2017 | 45 | 2017.03.06 | 2017.12.05 | 5270 | newborn |
| 425 | 2017 | 45 | 2016.12.12 | 2017.11.14 | 4724 | yearling |
| 436 | 2017 | 45 | 2016.11.15 | 2018.01.02 | 4823 | yearling |
| 465 | 2017 | 45 | 2017.05.27 | 2017.11.14 | 3335 | yearling |
| 451 | 2017 | 45 | 2017.03.22 | 2017.12.09 | 5842 | newborn |
| 445 | 2017 | 45 | 2017.03.30 | 2017.11.07 | 3743 | yearling |
| 471 | 2017 | 45 | 2017.06.17 | 2018.01.02 | 3435 | newborn |
