# Supplementary material for: Black bears alter movements in response to anthropogenic features with time of day and season
Source: Mov Ecol. 2019 Jul 11;7:19. doi: 10.1186/s40462-019-0166-4 (PMC6621962; doi:10.1186/s40462-019-0166-4)

**Additional File 2**

We evaluated two-state and three-state hidden Markov models with the R package moveHMM. The three-state models outperformed the two-state models. The three states can be interpreted as an encamped state, a foraging state, and a movement state.

**Figure S2.** Example results from a three-state hidden Markov model for a single bear. The three states identified were (1) encamped (orange), (2) foraging (blue), and (3) movement (green). S1a and S1b show the distribution of turning angles and step lengths. S1c shows the three behavioral states overlaid on the bear’s path.


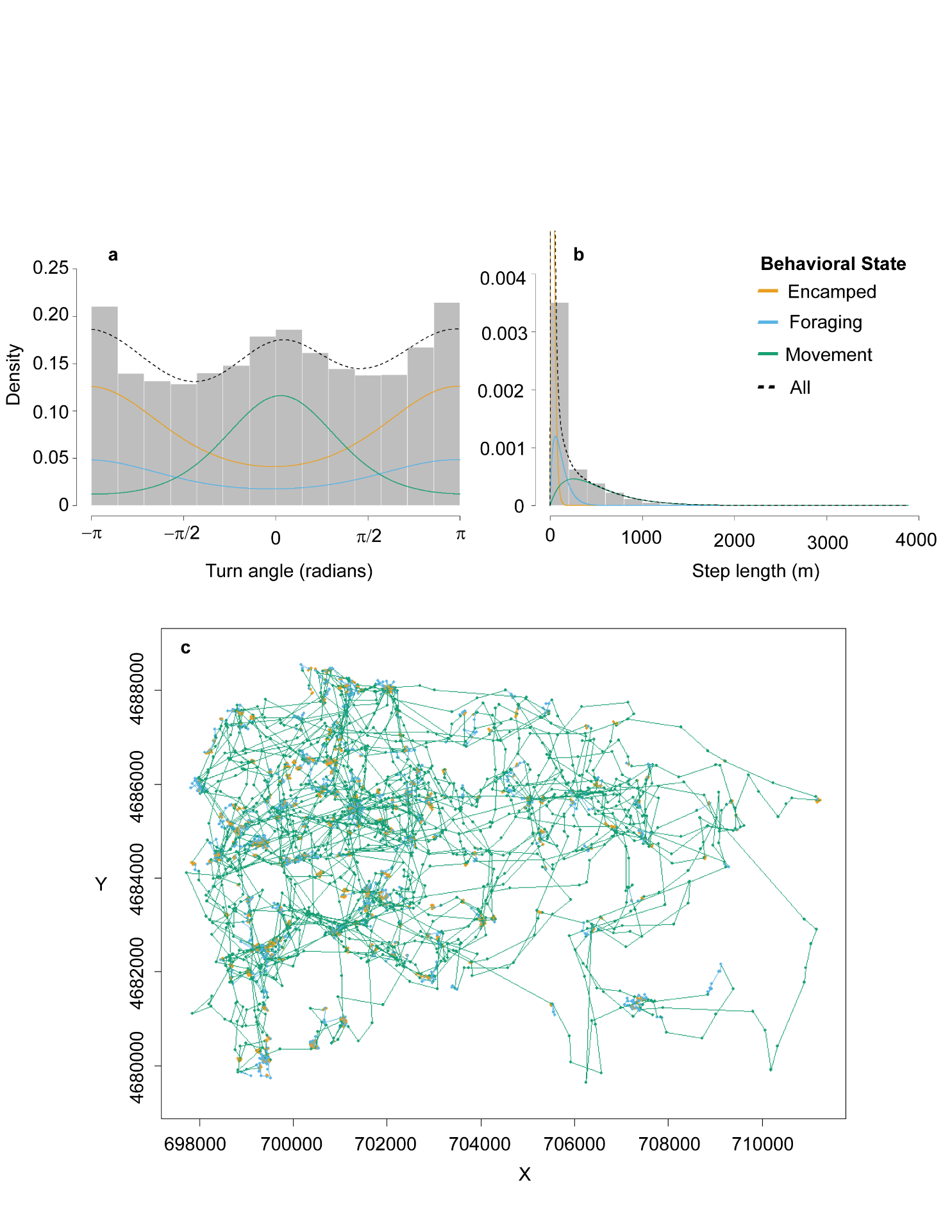

Supplement: Supplementary file 2 — Figure S2. Example results from a three-state hidden Markov model for a single bear. (DOCX 441 kb) [file 40462_2019_166_MOESM2_ESM.docx]
