# Supplementary material for: Black bears alter movements in response to anthropogenic features with time of day and season
Source: Mov Ecol. 2019 Jul 11;7:19. doi: 10.1186/s40462-019-0166-4 (PMC6621962; doi:10.1186/s40462-019-0166-4)
Supplement: Supplementary file 3 — Kernel Density Home Range Estimation (DOCX 14 kb) [file 40462_2019_166_MOESM3_ESM.docx]

**Additional File 3.** Kernel Density Home Range Estimation

To estimate black bear home ranges, we followed the recommendations of Borger et al. (2006), Bauder et al. (2015), and Fieberg (2007). First, to reduce home range estimation bias, we standardized our sampling among individuals by subsetting the collar data to every 6 hours. We used the ks (Duong 2018) package in the R software environment (R Core Team 2017) with the reference bandwidth and an unconstrained bandwidth matrix to estimate kernel density home ranges. The unconstrained bandwidth matrix has been shown to be less sensitive to fix rate and sampling duration than other home range estimators while producing a single volume contour (Bauder et al. 2015). We estimated kernel density home ranges for each season and identified the final home range boundaries as 95% of the entire utilization distribution for each individual.

**References**

Borger L, Franconi N, de Michele G, Gantz A, Meschi F, Manica A, et al. Effects of sampling regime on the mean and variance of home range size estimates. J Anim Ecol. 2006; 75: 1393±1405. https://doi.org/10.1111/j.1365-2656.2006.01164.x PMID: 17032372

Bauder JM, Breininger DR, Bolt MR, Legare ML, Jenkins CL, McGarigal K. The role of the bandwidth matrix in influencing kernel home range estimates for snakes using VHF telemetry data. Wildlife Research. 2015; 42: 437±453.

Duong T. 2018. ks: Kernel Smoothing. R package version 1.11.2. Available from https://CRAN.R-project.org/package=ks.

Fieberg J. Kernel density estimators of home range: smoothing and the autocorrelation red herring. Ecology. 2007; 88: 1059±1066. PMID: 17536721

R Core Team. 2017. R: A Language and Environment for Statistical Computing. R Foundation for Statistical Computing, Vienna, Austria. https://www.R-project.org
