# Supplementary material for: Black bears alter movements in response to anthropogenic features with time of day and season
Source: Mov Ecol. 2019 Jul 11;7:19. doi: 10.1186/s40462-019-0166-4 (PMC6621962; doi:10.1186/s40462-019-0166-4)

**Additional File 4**

**Figure S4**: Stacked bar plots of the proportion of individual black bear movement models with each spatial scale. Spatial scales ranged from fine (650 m) to coarse (1523 m) and were selected by running univariate models and identifying the scale with the lowest AIC value.
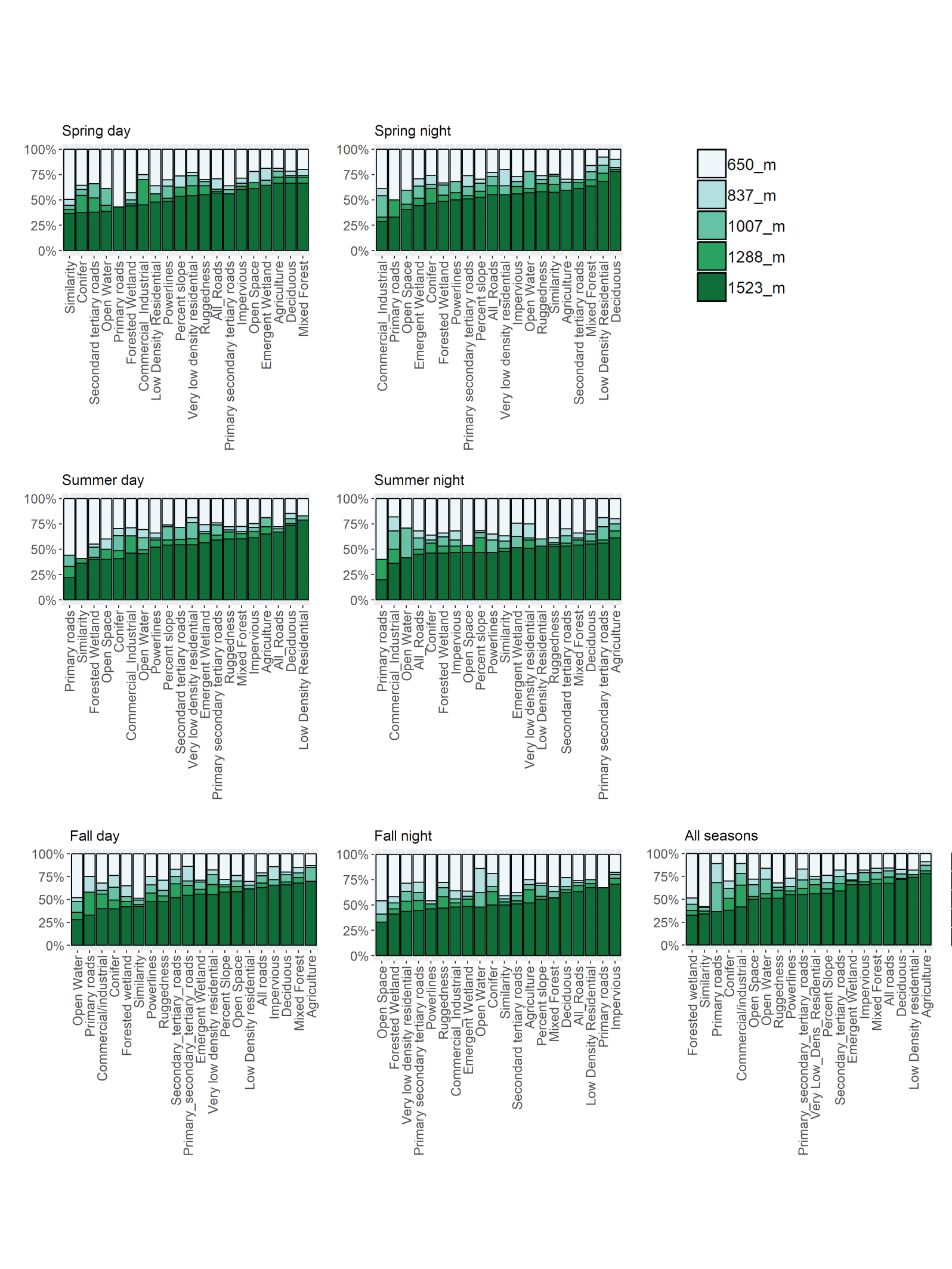

Supplement: Supplementary file 4 — Figure S4: Spatial scales of selection for black bear step selection functions. (DOCX 655 kb) [file 40462_2019_166_MOESM4_ESM.docx]
