# Supplementary material for: Black bears alter movements in response to anthropogenic features with time of day and season
Source: Mov Ecol. 2019 Jul 11;7:19. doi: 10.1186/s40462-019-0166-4 (PMC6621962; doi:10.1186/s40462-019-0166-4)

**Additional File 5**

**Figure S5:** Relative probability of black bear movement across the state of Massachusetts.


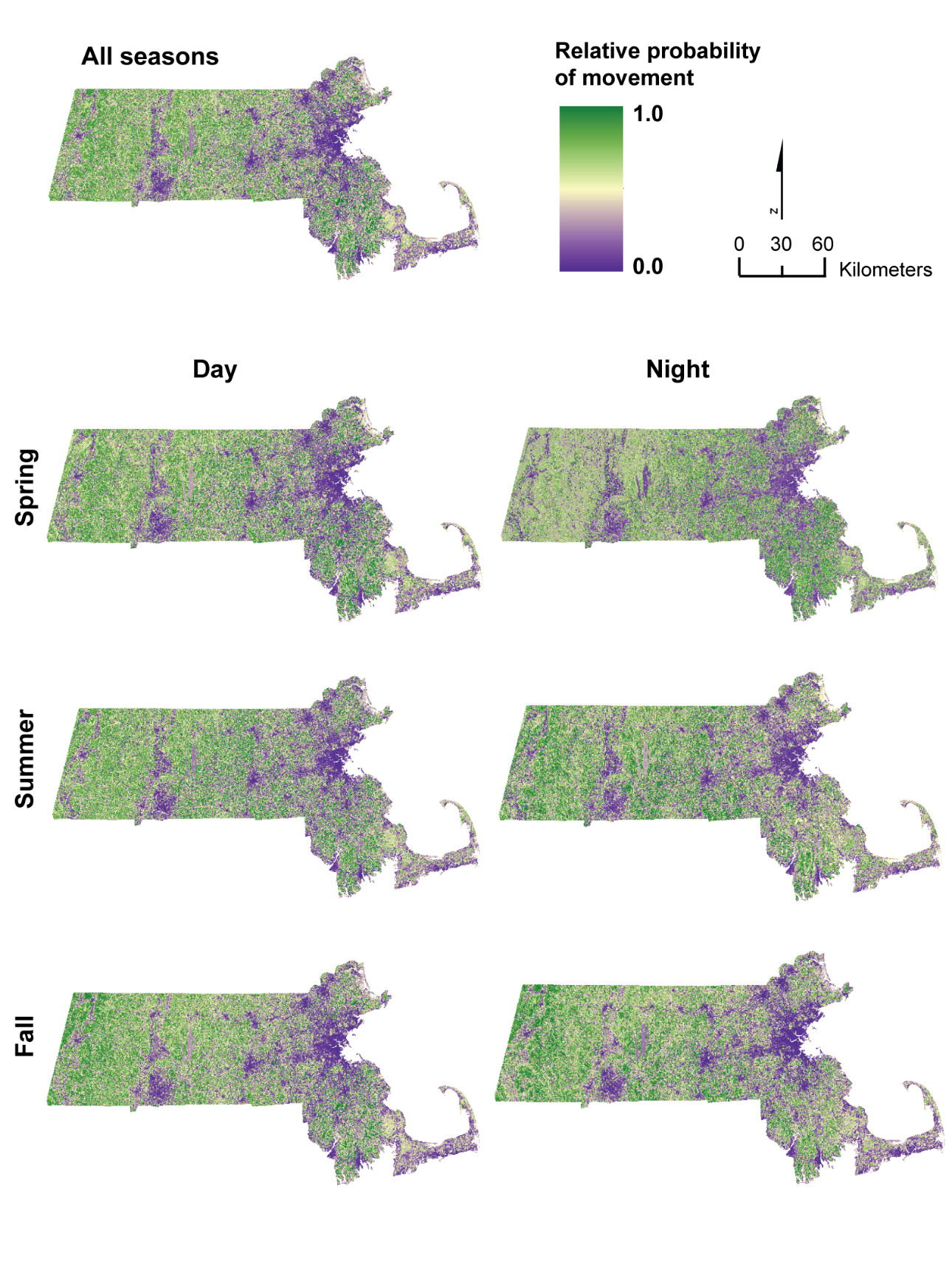

Supplement: Supplementary file 5 — Figure S5: Relative probability of black bear movement across the state of Massachusetts (DOCX 1689 kb) [file 40462_2019_166_MOESM5_ESM.docx]
